# Beyond memory: leveraging CSF biomarkers in all MCI patients ahead of anti-amyloid therapies

**DOI:** 10.1007/s10072-025-08250-3

**Published:** 2025-05-23

**Authors:** Martina Poli, Chiara Giuseppina Bonomi, Luca Tretola, Marta Rosa, Martina Gaia Di Donna, Alessandra Moreschini, Marzia Nuccetelli, Sergio Bernardini, Nicola Biagio Mercuri, Alessandro Martorana, Caterina Motta

**Affiliations:** 1 Memory Clinic and Neurodegenerative Dementia Research Unit, University of Rome Tor Vergata, Rome, Italy; 2 Division of Clinical Biochemistry and Clinical Molecular Biology, University of Rome Tor Vergata, Rome, Italy; 3 Neurology Unit, Policlinico Tor Vergata, University of Rome Tor Vergata, Rome, Italy

**Keywords:** Alzheimer’s disease, Mild cognitive impairment, Anti-amyloid monoclonal antibodies, Biomarkers

## Abstract

In the era of anti-amyloid (Aβ) monoclonal antibodies (AMABs), with the recent marketing authorization granted for lecanemab by European Medicines Agency (EMA), randomized clinical trial selection criteria typically include memory impairment detection. In a cohort of patients with Mild Cognitive Impairment (MCI), we observed a non-biunivocal relationship between Aβ pathology and memory deficits, with Aβ pathology present even in patients with atypical clinical presentations. This suggests that using MCI, regardless of the presence of memory impairment, as the sole general criteria along with amyloid pathology biomarkers, could expand the pool of eligible patients. The presence of patients with non-amnestic MCI and underlying Alzheimer’s Disease (AD) pathology makes a timely biomarker-based diagnosis necessary, as they may be considered eligible for treatment with AMABs according to current prescribing guidelines, even in the absence of memory impairment. However, it remains unclear whether, for such patients, the potential efficacy of AMABs actually outweighs the risks associated with treatment.

## Introduction

The advent of anti-amyloid (Aβ) monoclonal antibodies (AMABs) is changing the perspectives for the treatment of Alzheimer’s Disease (AD) [1]. The approval of lecanemab (Leqembi, Biogen)– a humanized IgG1 monoclonal antibody targeting Aβ protofibrils– by the Food and Drug Administration and the Medicines and Healthcare products Regulatory Agency in the United Kingdom, more recently followed by its marketing authorization by the European Medicines Agency, represent an important step forward, alongside the approval of donanemab (Kisunla, Lilly) in the United States for early symptomatic AD. Indeed, the forthcoming availability of these drugs is prompting clinicians throughout Europe to consider which workflow to adopt in order to provide as many patients as possible with access to disease-modifying treatments.

Envisaging a direct translation of the strict selection criteria used in randomized clinical trials (RCTs) in clinical practice, many patients that could potentially benefit from these drugs might be denied a prescription. Indeed, several research groups have used RCTs’ criteria to assess the proportion of patients eligible for AMABs in their cohorts, concluding that the percentage of individuals with early AD who qualify for these treatments is limited [2, 3].

So far, AMABs have been studied on patients with Mild Cognitive Impairment (MCI) and mild AD dementia, defined as progressive memory deficits and altered biomarker-based testing demonstrating AD pathology– Aβ positron emission tomography (PET) or cerebrospinal fluid (CSF) analysis [4]. A first-line use of neuropsychological tests for the detection of episodic memory impairment was applied in RCTs to identify an ultra-selected homogeneous population to be addressed to second-line assessments and eventual therapy administration, based on the idea that amnestic presentation and amyloid pathology are strictly intertwined [5].

According to this workflow, clinicians might be inclined to prioritize the confirmation of amyloid pathology in patients with amnestic syndrome, potentially overlooking those with atypical clinical presentations of AD who could also be treated with AMABs, in line with current indications.

The aim of the present observational retrospective study is to assess what relationship exists between memory and CSF evidence of amyloid pathology within our single-center cohort. This could be important to evaluate if amnestic impairment should be the bottleneck that determines how early and whether biomarker assessment should be performed, looking at the forthcoming need of selecting patients eligible for AMABs.

## Materials and methods

### Enrolment of study participants

**Fig. 1:**
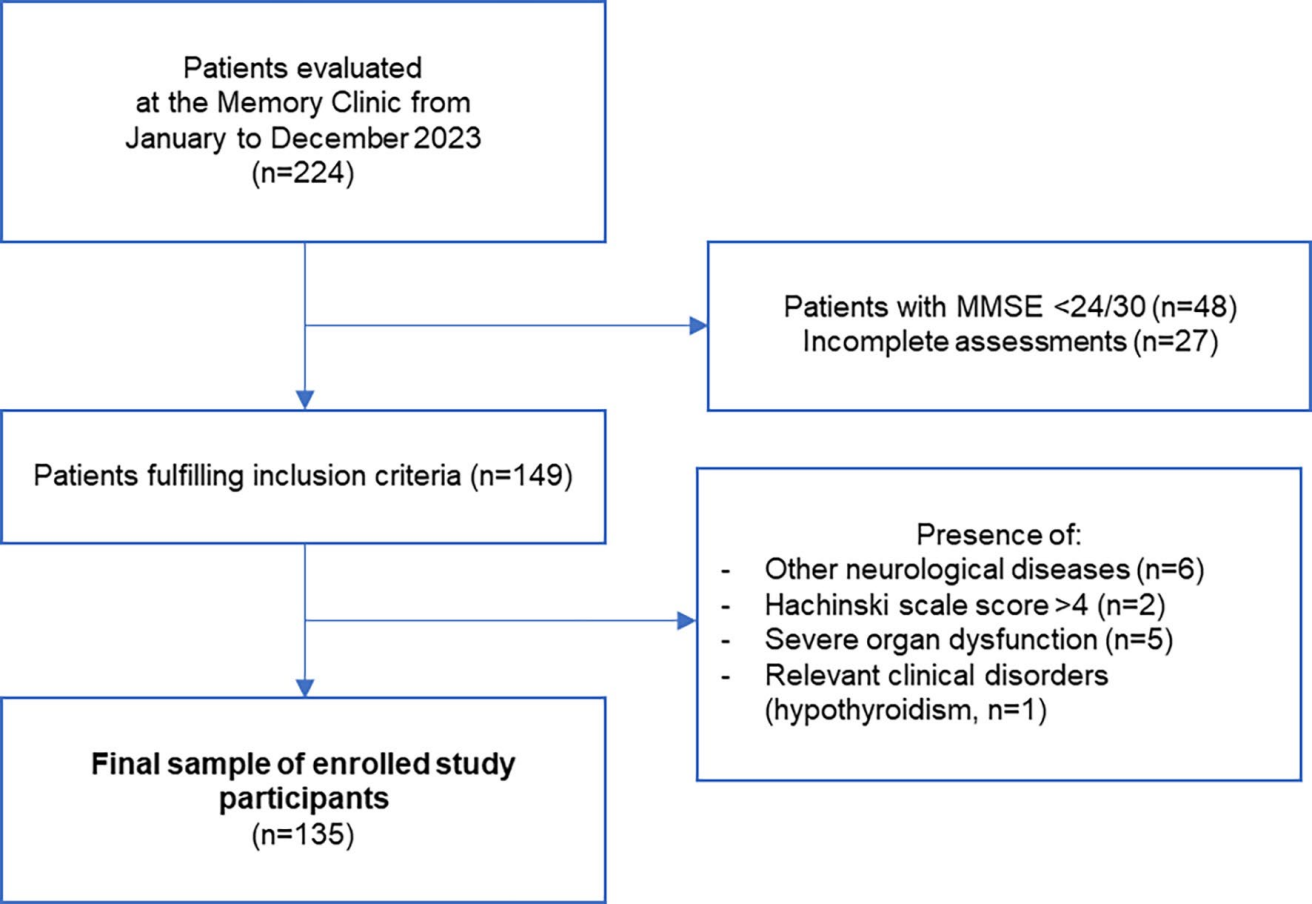
Flowchart summarizing the enrolment of study participants

We evaluated 224 patients referring to the Memory Clinic of the University Hospital “Policlinico Tor Vergata” between January and December 2023. The criteria for retrospective inclusion were: (1) availability of a complete neuropsychological assessment, laboratory testing, brain magnetic resonance imaging, 18-F-fluorodeoxyglucose-PET, lumbar puncture; (2) fulfillment of the diagnostic criteria for MCI [6]. Exclusion criteria were: (1) other primary neurological diseases (e.g. neuroinfectious diseases, traumatic brain injury, Parkinson’s Disease, Multiple Sclerosis); (2) Hachinski scale score > 4 at baseline MRI, held suggestive of vascular co-pathology; (3) major comorbidities such as severe organ dysfunction; (4) evidence of other clinical disorders that could affect the accuracy of the data (Fig. 1).

We eventually selected 135 patients, who all had undergone the following neuropsychological battery [7]: Mini Mental State Examination (MMSE) to assess global cognitive functions; Rey Auditory Verbal Learning Test (RAVLT) immediate and delayed recall for verbal episodic memory; Rey-Osterrieth Complex Figure Test copy and recall for visuo-spatial memory; Raven Colored Progressive Matrices for abstract reasoning; Stroop test and verbal fluency test for attention and executive functions. Patients were stratified into three major clinical subtypes according to cognitive profiles emerging from the neuropsychological evaluation, considering the presence or absence of memory impairment as well as the involvement of one single domain versus multiple-domains, as reported elsewhere [8]: MCI with single-domain amnestic impairment in episodic memory (aMCI-SD, *n* = 21), defined as impairment in RAVLT immediate and delayed or delayed recall alone, as the latter was considered the most sensitive test for episodic memory in agreement with the neuropsychologist’s evaluation; multiple domain MCI including amnestic impairment (aMCI-MD, *n* = 68); multiple domain MCI without amnestic impairment (naMCI-MD, *n* = 46). Since no patient met the criteria for the definition of non-amnestic MCI single domain, we omitted that category.

All procedures were performed according to the Declaration of Helsinki. All patients/legal representatives signed an informed consent for the procedures carried out in the study.

The study protocol was approved by the local Ethics Committee (approval number 57.25CET2PTV).

### CSF, blood sampling and biomarkers analysis

All lumbar punctures and CSF biochemical analyses for MCI patients were performed according to standard practice as described elsewhere [9]. The samples were centrifuged at 3000 g at + 4 °C for 10 min, aliquoted and frozen at -80 °C.

Levels of CSF Aβ_40_, Aβ_42_ and phosphorylated tau 181 (p-tau181) as well as of CSF total tau (t-tau) were measured using fully-automated CLEIA Fujirebio LUMIPULSE^®^ G1200 (Fujirebio, Inc., Tokyo, Japan). The cut-off points used to define the presence of amyloid pathology (A status) and of tau pathology (T status) in the CSF were as follows: CSF Aβ_42_/Aβ_40_ < 0.0667 (A+); CSF p-tau181 > 56 pg/mL (T+).

### Data analysis

We visualized the flow from the three neuropsychological categories of MCI (aMCI-SD, aMCI-MD, naMCI-MD) to different possible CSF profiles (A + T+, A + T-, A-T + and A-T-) through a Sankey Diagram, in which the width of the arrows is drawn proportional to the amount of flow (SankeyMATIC, https://sankeymatic.com). All continuous variables, including levels of CSF biomarkers and neuropsychological tests scores, were expressed as means ± standard deviations. To evaluate differences between groups, the analysis of variance (ANOVA) and post-hoc Bonferroni corrections for multiple comparisons of continuous variables were performed using JASP (JASP, Version 0.14.1, Computer software, https://jasp-stats.org). The differences between groups frequency of A + status and neuropsychological tests expressed as categorical variables across MCI categories were assessed with χ-squared test performed in GraphPad Prism (GraphPad Software, Boston, Massachusetts, USA, www.graphpad.com). Values of *p* < 0.05 were considered statistically significant.

## Results

Our cohort included patients with aMCI-SD (*n* = 21, 15.6%), aMCI-MD (*n* = 68, 50.4%) and naMCI-MD (*n* = 46, 34%). Clinical and demographic characteristics of MCI patients, and p-values from ANOVAs between groups are reported in Table 1. Specifically, groups differed in age with aMCI-MD patients being younger than naMCI-MD (p_bonferroni_=0.024), while no differences in sex prevalence were found. Looking at CSF AD biomarkers levels, we retrieved significantly lower values of CSF Aβ42/Aβ40 in aMCI-SD and in aMCI-MD with respect to naMCI-MD (both p_bonferroni_<0.001), while higher levels of both CSF p-tau and CSF t-tau were found in aMCI-MD with respect to naMCI-MD (p_bonferroni_=0.014 and p_bonferroni_=0.028 respectively). As expected, significant differences were also observed in neuropsychological assessments across groups (see Table 1).

**Table 1:** Demographics, clinical and laboratory characteristics across patients with different neuropsychological profiles

	aMCI-SD (*n* = 21)	aMCI-MD (*n* = 68)	naMCI-MD (*n* = 46)	*p*-value
Age (yo)	70.48 ± 6.19	70.19 ± 5.95	73.11 ± 4.99	**0.024** ^**a, c**^
Sex (F%)	52.4%	51.5%	56.2%	0.868
CSF Aβ42/Aβ40	**0.03 ± 0.01**	**0.04 ± 0.01**	**0.06 ± 0.02**	**< 0.001** ^**b, c**^
CSF p-tau (pg/ml)	74.1 ± 32.38	83.07 ± 41.51	63.07 ± 29.76	**0.018** ^**c**^
CSF t-tau (pg/ml)	488.57 ± 213.57	592.09 ± 306.03	454.57 ± 243.72	**0.026** ^**c**^
MMSE	25.12 ± 1.15	25.06 ± 1.37	26.21 ± 1.61	**< 0.001** ^**b, c**^
RAVLT immediate	27.13 ± 6.63	21.25 ± 7.30	29.12 ± 9.72	**< 0.001** ^**a, c**^
RAVLT immediate (pathological%)	52.4%	83.8%	43.5%	**< 0.001** ^**a, c**^
RAVLT delayed	1.5 ± 1.46	1.57 ± 1.51	5.95 ± 1.57	**< 0.001** ^**b, c**^
RAVLT delayed (pathological%)	100%	100%	0%	**< 0.001** ^**b, c**^
Rey figure copy	28.24 ± 3.90	18.82 ± 10.09	21.23 ± 7.58	**< 0.001** ^**a, b**^
Rey figure copy (pathological%)	0%	44.1%	19.6%	**< 0.001** ^**a, b,c**^
Rey figure delayed	8.79 ± 6.63	7.85 ± 6.58	21.85 ± 5.05	**< 0.001** ^**b, c**^
Rey figure delayed (pathological%)	95.2%	94.1%	76.1%	**0.008** ^**b, c**^
Stroop test	22.36 ± 14.31	51.17 ± 30.76	35.71 ± 21.48	**< 0.001** ^**a, c**^
Stroop test (pathological%)	0%	64.7%	45.7%	**< 0.001** ^**a, c**^
Raven matrices	25.75 ± 6.56	20.64 ± 7.67	23.98 ± 4.85	**< 0.001** ^**a, c**^
Raven matrices (pathological%)	0%	33.8%	4.3%	**0.008** ^**a, b,c**^
Verbal fluency	33.02 ± 5.52	18.89 ± 8.6	24.42 ± 9.18	**< 0.001** ^**a, b,c**^
Verbal fluency (pathological%)	0%	76.5%	67.4%	**< 0.001** ^**a, b**^

*Legend*. The data are reported as mean ± standard deviation (SD), unless when otherwise specified. aMCI-SD: amnestic mild cognitive impairment single domain. aMCI-MD: amnestic mild cognitive impairment multiple domain. naMCI-MD: non-amnestic mild cognitive impairment multiple domain. CSF: cerebrospinal fluid. P-tau: phosphorylated tau 181. T-tau: total tau. MMSE: Mini-Mental State Examination. RAVLT: Rey Auditory Verbal Learning Test

a = post hoc *p* < 0.05 aMCI-SD vs. aMCI-MD

b = post hoc *p* < 0.05 aMCI-SD vs. naMCI-MD

c = post hoc *p* < 0.05 aMCI-MD vs. naMCI-MD

**Fig. 2:**
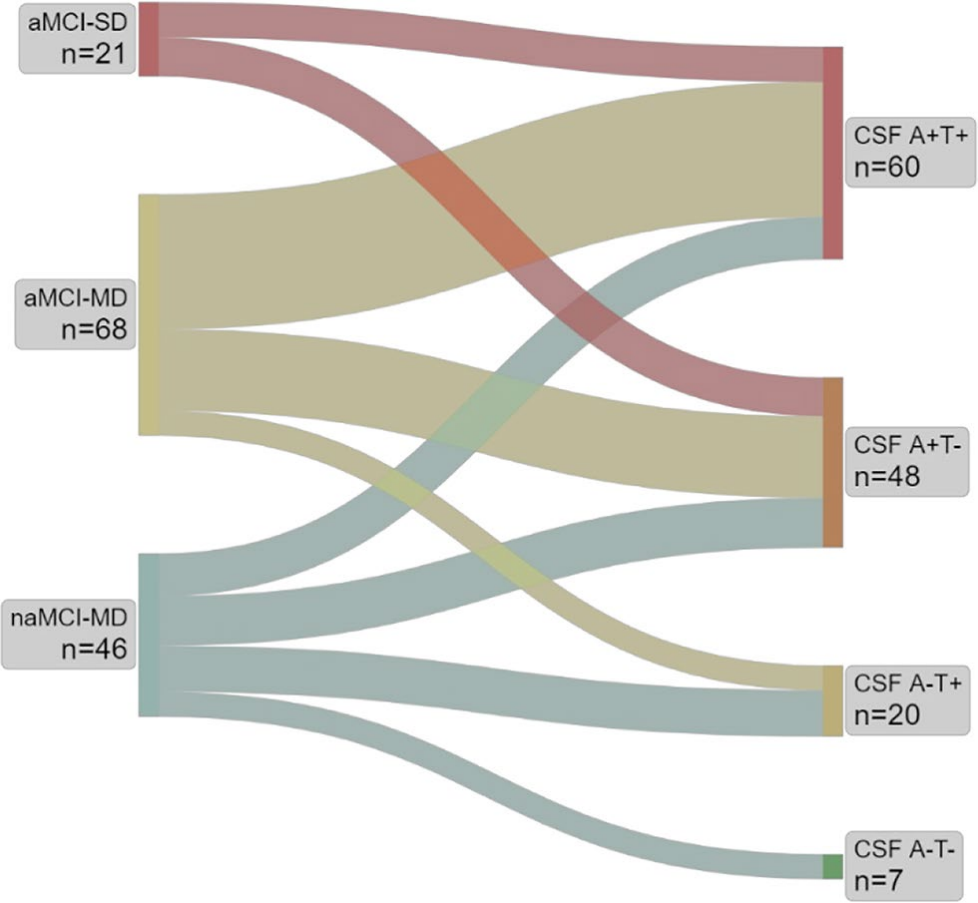
Sankey diagram illustrating cerebrospinal fluid Alzheimer’s Disease biomarkers of patients with different neuropsychological profiles. *Legend.* aMCI-SD: amnestic mild cognitive impairment-single domain; aMCI-MD: amnestic mild cognitive impairment-multiple domain; naMCI-MD: non-amnestic mild cognitive impairment-multiple domain. CSF: cerebrospinal fluid

As represented in Fig. 2, **100**% of patients with aMCI-SD showed CSF A + status, with 52.4% of them having isolated Aβ pathology (A + T-: *n* = 11) and 47.6% showing increased p-tau181 levels (A + T+: *n* = 10). In aMCI-MD patients A + status was found in 89.7% cases (A + T-: *n* = 23, 33.8%; A + T+: *n* = 38, 55.9%), 10.3% presented with an A-T + profile (*n* = 7) and none showed normal AD biomarkers (A-T-). Finally, the naMCI-MD group showed the highest variability of CSF profiles, with A + found in 56.4% of cases (A + T-: *n* = 14, 30.4%; A + T+: *n* = 12, 26%), A-T + profile in 28.2% (*n* = 13), and normal biomarkers in 15.2% of patients (*n* = 7). The Chi-squared test confirmed a different frequency of A + status across the three MCI categories (*p* < 0.001), while the frequency of T + in A + patients was comparable between groups [χ2 (2) = 2.589, *p* = 0.274)]. Chi-squared analyses also revealed significant differences between groups in all neuropsychological test administered, namely RAVLT immediate (χ2 (2) = 21.48, *p* < 0.001) and delayed recall (χ2 (2) = 135.00, *p* < 0.001), Rey Figure copy (χ2 (2) = 18.15, *p* < 0.05) and delayed recall (χ2 (2) = 9.74, *p* = 0.008), Stroop test (χ2 (2) = 27.08, *p* < 0.001), Raven’s matrices (χ2 (2) = 9.76, *p* = 0.008), and verbal fluency (χ2 (2) = 40.65, *p* < 0.001).

Overall, the copresence of amnestic syndrome and A + status was found in 82 patients out of our cohort of 135 patients with MCI (60.7%). Conversely, A + status, regardless of neuropsychological profile, was found in 108 patients (80%).

## Discussion

With this study we aimed to evaluate how CSF AD biomarker changes unfold according to clinical presentation, and whether combining early biomarker assessment with neuropsychological evaluation could help identifying a higher number of patients likely to benefit from AMABs.

Considering the correspondence between these neuropsychological categories and CSF biomarker changes, we first observed that the condition of aMCI-SD was invariably associated with Aβ pathology (100%), confirming a virtually perfect concordance with an A + status. When amnestic impairment was present in the context of multiple domain impairment (aMCI-MD), the prevalence of Aβ pathology was also very high, as it was detected in almost 90% of patients. Instead, in the absence of memory deficit (naMCI-MD) A + status was found only in around 60% of patients, who still represent the majority within this category. Interestingly, the prevalence of Aβ pathology was different across the three MCI categories, while tau pathology (T+) was found in equal proportions whenever an A + status was confirmed. This is consistent with the notion that the presence of Aβ pathology primarily leads to the onset of memory dysfunction, typical of AD, while tau pathology might be more linked to disease progression [10].

Moreover, in our cohort we found an A-T + status in 20 patients (15%), all belonging to the multidomain MCI categories (aMCI-MD and naMCI-MD), while only 7 patients (5%) showed normal CSF biomarkers (A-T-). Both these CSF profiles are held indicative of non-AD pathology, and the clinical progression of these subjects should be carefully evaluated considering other possible forms of dementia [4].

These results show a high concordance between amnestic syndrome and Aβ pathology [11], which in turn may also underscore other neuropsychological phenotypes not including or not limited to memory impairment. In our sample, these patients constitute approximately one-third of A + subjects, suggesting that biomarkers assessment may expand the cohort that could be addressed to AMABs. The increase in this percentage– from 60 to 80%– seems crucial considering that exclusion criteria could further narrow the eligible population. On the other hand, clinical studies have not been conducted in non-amnestic patients with amyloidopathy, who are hypothetically encompassed within the drug approval criteria. This raises challenges in predicting the efficacy and potential side effects in this subgroup and, notably, it remains unclear whether the favorable benefit-to-risk ratio that led to the approval of AMABs is confirmed in this population.

This study has limitations, for instance the sample size which affected the proportion of patients within the single MCI subgroups. In particular, the number of patients with aMCI-SD was lower with respect to the other two categories, potentially impacting the results of the analyses. Broader evaluations should be performed, considering the limitations coming from the restricted time-window that we applied to our retrospective study. Longitudinal data would also clarify the clinical progression of patients with atypical presentations and indeterminate CSF biomarkers. Furthermore, following the very recent approval of lecanemab in the European Union, it should be appropriate to evaluate how a real-world cohort aligns with the full treatment indication outlined in the Summary of the Product Characteristics (“adult patients with a clinical diagnosis of mild cognitive impairment and mild dementia due to Alzheimer’s disease (Early Alzheimer’s disease) who are apolipoprotein E ε4 (ApoE ε4) non-carriers or heterozygotes with confirmed amyloid pathology”). In this regard, our study only focused on patients with MCI, not including individuals with mild AD dementia. Additionally, ApoE genotyping was not systematically available for all patients, limiting our ability to stratify the sample accordingly. Nonetheless, our findings reveal how the choice of selection criteria—particularly when accounting for non-amnestic clinical profiles—can significantly affect the proportion of individuals potentially eligible for treatment with AMABs.

In conclusion, in a hypothetical workflow designed to optimize the prescription of AMABs, we suggest that biomarker assessment not subordinated to the presence of memory deficits could expand the pool of patients eligible according to current guidelines. Conversely, this approach would include patients without clear memory impairment, who were not evaluated in the initial clinical trials, and for whom it is not yet possible to determine whether the benefits outweigh the risks. Further research is needed to confirm the clinical benefits across different neuropsychological profiles. Additionally, while the presence of overt cognitive decline is currently held pivotal to consider treatment, growing evidence advocates that the benefit of AMABs could be even greater in the earliest stages of AD. This is particularly relevant in the era of blood-based biomarkers, that as soon as clinically available would provide a less invasive and reliable method for early diagnosis and for the prediction of clinical progression.

## Data Availability

The data supporting the findings of this study is available on request from the corresponding author. The data is not publicly available due to privacy or ethical restrictions.
